# Einseitige Optikusatrophie bei einem 9-jährigen Patienten

**DOI:** 10.1007/s00347-020-01147-w

**Published:** 2020-07-01

**Authors:** Anna Nguyen-Höhl, Mohammed H. Khattab, Marco Henneke, Nicolas Feltgen, Hans Hoerauf, Sebastian Bemme

**Affiliations:** 1 Klinik für Augenheilkunde, Universitätsmedizin Göttingen, Göttingen, Deutschland; 2 Klinik für Kinder- und Jugendmedizin, Universitätsmedizin Göttingen, Göttingen, Deutschland

**Keywords:** Chorioretinitis, Vitritis, Optikusatrophie, Visusminderung, Skotom, Chorioretinitis, Vitritis, Optic atrophy, Loss of visual acuity, Scotoma

## Abstract

Ein 9‑jähriger Patient stellte sich aufgrund einer seit 2 Wochen zufällig bemerkten Sehminderung auf dem linken Auge vor. Funduskopisch waren neben einer leichten Vitritis eine blasse Papille, enggestellte Gefäße und eine Reflexvermehrung der Netzhautoberfläche sichtbar. In der Blutuntersuchung waren die Werte bis auf eine Eosinophilie und einen erhöhten Immunglobublin E(IgE)-Antikörpertiter normal.

## Abstract (en)

A 9‑year-old patient presented with a reduction of visual acuity in the left eye, which was incidentally noticed 2 weeks previously. Funduscopy revealed a mild vitritis, a pale optic disk, narrowed blood vessels and an increased reflex of the retinal surface. The values in the blood examination were normal apart from eosinophilia and an elevated immunoglobulin E (IgE) antibody titer.

## Anamnese

Ein 9‑jähriger Patient wurde mit der Verdachtsdiagnose eines Astarterienverschlusses und einer Optikusatrophie am linken Auge in unserer Poliklinik vorgestellt. Der Patient berichtete uns von einer zufällig bemerkten Sehverschlechterung seit ungefähr 2 Wochen. Eine ophthalmologische Untersuchung habe vor Eintreten der Beschwerden noch nicht stattgefunden. Augenverletzungen oder Augenoperationen wurden verneint, bisherige Augenerkrankungen waren nicht bekannt. Die Familienanamnese war unauffällig. Die Mutter berichtete, dass die Familie bis zum 3. Lebensjahr des Kindes in Südamerika lebte.

## Klinischer Befund

**Figure 1:**
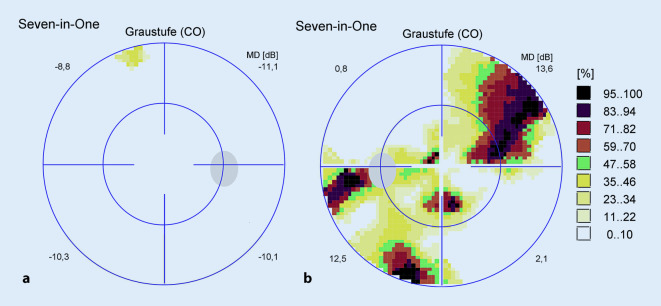

**Figure 2:**
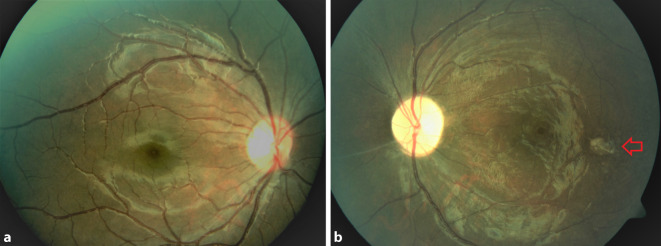

**Figure 3:**
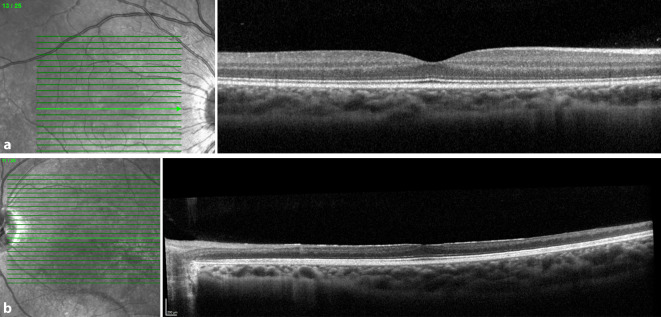

**Figure 4:**
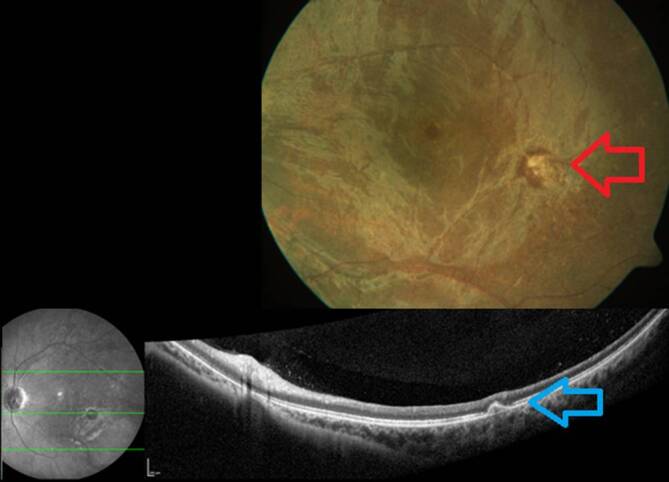

Der unkorrigierte Visus in der Ferne betrug 1,0 rechts und 0,05 links. Der Nahvisus lag rechts bei 1,0 und links bei 0,1. Sowohl mit Korrektion als auch mit stenopäischer Lücke war kein besserer Visus zu erheben. Die Augenmotilität war beidseits frei. Es bestanden keine Augenbewegungsschmerzen. Die Pupillomotorik auf dem rechten Auge war unauffällig, auf dem linken Auge war ein relativer afferenter Pupillendefekt (RAPD) nachweisbar. Das 30°-Gesichtsfeld am rechten Auge war frei (Abb. 1a), am linken Auge fanden wir unspezifische Skotome (Abb. 1b). Der vordere Augenabschnitt war beidseits unauffällig und reizfrei. Der Glaskörper rechts war unauffällig, links sahen wir eine milde zelluläre Infiltration. Der Augenhintergrund des rechten Auges war unauffällig (Abb. 2a). Am linken Auge sahen wir eine randscharfe aber blasse Papille, einen fehlenden Wallreflex der Makula, enggestellte Gefäße und eine fleckige Reflexvermehrung der gesamten Netzhautperipherie (Abb. 2b). Zudem war temporal der Makula eine gelbliche Läsion zu sehen. Ähnliche Läsionen fanden sich auch vereinzelt in der mittleren Netzhautperipherie. In der Spectral-Domain-optischen Kohärenztomographie (SD-OCT) waren die zentrale und mittelperiphere Netzhaut im Vergleich zum rechten Auge deutlich verdünnt bzw. atrophiert (Abb. 3a, b). Die gelbliche Läsion temporal der Makula stellte sich in der SD-OCT als hyperreflektive Ablagerung unter dem retinalen Pigmentepithel (RPE) dar (Abb. 4), und der präretinal anliegende Glaskörper war zellig infiltriert.

## Diagnostik

Aufgrund der einseitigen Glaskörperinfiltration wurde eine Uveitisbasisdiagnostik durchgeführt. Die Testung auf Borrelien, Lues, *Toxocara, Bartonella henselae* und Toxoplasmose waren nicht wegweisend. Auch Rheumafaktor, antinukleäre Antikörper und HLA-B51 waren negativ. Zur weiteren Diagnostik bei einseitiger Optikusatrophie führten wir zudem eine Magnetresonanztomographie des Schädels und der Orbita (cMRT) durch, der Befund war unauffällig. Die anamnestische Besonderheit unseres Patienten bestand darin, dass er bis zum 3. Lebensjahr in Südamerika lebte. Wegen des einseitigen subakuten Verlaufs mit diffuser Netzhautbeteiligung bei vorliegender entzündlicher Genese (Vitritis) einerseits und zunächst unauffälligem Uveitislabors andererseits, stellten wir die Verdachtsdiagnose einer diffusen unilateralen subakuten Neuroretinitis (DUSN). Auch die bei unserem Patienten sichtbare Optikusatrophie, Gefäßobliteration, fleckige Reflexvermehrung der Netzhautoberfläche („Oréfice’s sign“) und gelbliche Sub-RPE-Ablagerungen sind typische Befunde bei dieser Erkrankung. Die erweiterte Diagnostik mit Nachweis einer Eosinophilie im Differenzialblutbild und erhöhter IgE-Antikörper ohne bekannte Atopie bzw. Allergien untermauerte schließlich die Diagnose.

## Wie lautet Ihre Diagnose?

## Therapie und Verlauf

Die DUSN ist eine multifokale Chorioretinitis, die durch Nematoden verursacht wird. Nach Rücksprache mit den Pädiatern unserer Klinik wurde eine Therapie mit Albendazol 400 mg 1‑mal täglich p.o. über 4 Wochen eingeleitet, zusätzlich wurde Prednisolon 1 mg/kg Körpergewicht täglich p.o. verordnet und im Verlauf schrittweise abdosiert. Da bei unserem Patienten ein Fadenwurm funduskopisch nicht sichtbar war, kam eine interventionelle Behandlung mittels fokaler Laserkoagulation (LK) oder eine chirurgische Entfernung des Nematoden mittels Vitrektomie nicht infrage. Bei unserem Patienten lag bereits eine Spätform der Erkrankung mit Optikusatrophie vor, eine Visusbesserung konnte deshalb leider auch nach der Therapie nicht erreicht werden.

## Diskussion

Die diffuse unilaterale subakute Neuroretinitis wurde 1978 erstmalig von Gass beschrieben. Dabei handelt es sich um eine multifokale Chorioretinitis, verursacht durch im subretinalen Raum migrierende Nematoden [1]. Diese gelangen über einen Zwischenwirt wie Hunde oder Waschbären oder auf direktem Wege durch Verschlucken von Nematodeneiern in die Blutwege und letztendlich in das Auge [1]. Unter den Nematoden wurde zuerst *Toxocara canis* als Ursache für DUSN beschrieben [2]. Zu den ursächlichen Erregern sind neben *Baylisascaris procyonis* (ein Parasit, der Waschbären und Stinktiere befällt, häufiges Vorkommen im mittleren Westen der USA) und *Ancylostoma caninum* (ein Hunde-Hakenwurm, häufiges Vorkommen im Südosten der USA) noch einige weitere Nematoden zu zählen [1–4]. Auch in Deutschland sind bereits einige Fälle von DUSN beschrieben, als Erreger vermutet wurde *Baylisascaris procyonis* [5]. Letztendlich war bei unserem Patienten der Infektions-Zeitpunkt/-Ort nicht nachzuweisen. Sowohl eine frühe Infektion in den ersten Lebensjahren in Südamerika, als auch eine spätere Infektion in Deutschland ist denkbar. Die endemischen Orte schließen Lateinamerika und Deutschland ein, jedoch wird von häufigeren Fällen aus Lateinamerika berichtet [6, 7]. Des Weiteren infizieren sich v. a. Kinder < 2 Jahre mit den Nematodeneiern [1]. Die Larven können im Gewebe Monate bis Jahre überleben [1, 2]. Da die Erkrankungen abwechselnd akute und latente Phasen aufweisen, wird DUSN in vielen Fällen von den Betroffenen nicht bemerkt bis die Spätphase eintritt.

DUSN tritt vorwiegend unilateral auf, seltener wurden bilaterale Verläufe beschrieben [8]. Betroffen sind meist die gesamte Retina sowie das RPE, die retinalen Gefäße, der subretinale Raum, der Sehnerv und die Chorioidea. Vermutet werden eine durch Nematoden und deren Produkte verursachte Inflammation und Degeneration der äußeren Retinaschichten [2]. Die Klassifizierung der DUSN in Früh- und Spätphase erfolgt anhand der klinischen Erscheinung. Die Frühphase ist gekennzeichnet durch einen verminderten Visus, eine moderate Vitritis, Papillitis, retinale Vaskulitis und wiederkehrende gelb-weißliche äußere retinale und chorioidale Läsionen [1]. Die Spätphase dagegen ist durch Optikusatrophie, retinale Arterienverengung, Degenerationen der äußeren Retinaschichten mit diffusen RPE-Unterbrechungen und damit einhergehender erheblicher Visusminderung sowie einem RAPD charakterisiert [1, 2]. Funduskopisch sind vermehrte Reflexe der internen limitierenden Membran („Oréfice’s sign“) typisch.

Nur die fundoskopische Darstellung eines Fadenwurmes führt zur Diagnosesicherung. Nematoden können in jedem Stadium am Augenhintergrund aufgefunden werden. Der Fadenwurm zeigt sich dabei häufig beweglich. Abhängig von der Spezies beträgt die Fadenwurmlänge 400 bis zu 2000 µm. Oftmals ist der Fadenwurm jedoch nicht sichtbar, sodass indirekte je nach Früh- oder Spätphase typische Fundusveränderungen eine okuläre Beteiligung der Nematodeninfektion vermuten lassen. Subretinale Spuren (91,7 %), fokale Veränderungen des RPEs (89,3 %), kleine weißliche punktförmige Läsionen (80,2 %) und eine Optikusatrophie (76,9 %) sind dabei die häufigsten klinischen Zeichen [9]. In der SD-OCT zeigt sich häufig eine Atrophie der Netzhaut am hinteren Pol mit Ausdünnung der retinalen Nervenfaserschichtdicke und Aufhebung der fovealen Senke. Des Weiteren lassen sich in der SD-OCT gelegentlich subretinale Tunnel („Garcia’s sign“) erkennen, bzw. der Fadenwurm selbst kann als intraretinale Hyperreflektivität dargestellt werden [10]. Eine im Blut nachgewiesene Eosinophilie und IgE-Antikörper-Erhöhung weist auf eine parasitäre Infektion hin.

**Diagnose:** Diffuse unilaterale subakute Neuroretinitis (DUSN)

Als ergänzende Diagnostik kann eine Elektroretinographie (ERG) durchgeführt werden. Aufgrund des jungen Alters unseres Patienten wurde darauf verzichtet. Zu erwarten wäre dabei eine reduzierte B‑Welle proportional zur retinalen Beteiligung [11].

Differenzialdiagnostisch ist in der Frühphase aufgrund der fokalen Chorioretinitis an Sarkoidose, Toxoplasmose, Histoplasmose, multifokale Chorioiditis, serpinginöse Chorioiditis, akute posteriore multifokale plakoide Pigmentepitheliopathie, Multiple-Evanescent-White-Dot-Syndrom, unspezifische Optikusneuritis und Papillitis zu denken. Die Spätphase einer DUSN kann einer posttraumatischen Chorioretinopathie, okklusiven Gefäßerkrankung, Chorioretinitis bei Sarkoidose, Retinopathia pigmentosa, LHON oder toxischen Retinopathie [12, 13] ähneln. Bei Befall des zentralen Nervensystems wurden neuropsychiatrische und neurodegenerative Symptome, u. a. Schizophrenie, kognitive Defizite und demenzielle Erkrankungen, beobachtet [14]. Schwere Infektionen beim Kind können sich durch plötzliche Lethargie, Nackensteifigkeit, Torticollis, Opisthotonus, Ataxie, verminderte Kopfkontrolle, Verlust der Feinmotorik, Unfähigkeit alleine zu sitzen, stehen oder gehen äußern [14, 15].

Therapie der Wahl bei Darstellung des Fadenwurmes ist eine Laserkoagulation (LK) des Parasiten. Bei der LK wird der Fadenwurm durch konfluierende Laserherde von 200 µm Größe bei 200 mW und 0,2 s Dauer mit einem 532- oder 810-nm-Laser abgeriegelt, gefolgt von einer lückenlosen weiteren Umstellung. Befindet sich der Fadenwurm nahe der Fovea mit Gefahr einer bleibenden zentralen Sehminderung nach LK, können sehr zarte Laserherde appliziert werden, um den Fadenwurm Richtung mittlere Peripherie zu bewegen und anschließend dort die LK des Fadenwurmes durchzuführen [13]. Auch die Vitrektomie wurde als Therapieoption beschrieben [13]. In den meisten Fällen lässt sich aber der Fadenwurm nicht lokalisieren, da Nematoden von Licht wegmigrieren. In diesem Fall beschränkt sich die Behandlung auf eine systemische Therapie mit Antihelminthika, z. B. Albendazol [5]. Begleitend können Kortikosteroide eingesetzt werden, um eine antiinflammatorische Wirkung zu erzielen [16, 17]. Bei Kindern sollte die Therapie unbedingt in enger Abstimmung mit einem Kinderarzt erfolgen.

## Fazit für die Praxis

- DUSN ist eine multifokale Chorioretinitis, verursacht durch Nematoden.
- Typisch ist der einseitige subakute Verlauf mit Beteiligung der gesamten Netzhaut.
- Die Reiseanamnese kann bei der Diagnosestellung wegweisend sein.
- Diagnostisch hilfreich ist der Nachweis einer Eosinophilie und einer IgE-Erhöhung.
- Frühphase mit moderaten Entzündungszeichen (Vitritis, Papillitis, Vaskulitis).
- Spätphase mit Optikus- und Netzhautatrophie einhergehend mit einem RAPD.
- Therapie der Wahl ist die Laserkoagulation, sofern der Fadenwurm funduskopisch sichtbar ist.
- Alternativ kommen Antihelminthika (z. B. Albendazol) als systemische Therapie zum Einsatz.

## References

[1] Gass JDM, Gilbert WR, Guerry RK, Scelfo R (1978). Diffuse unilateral subacute neuroretinitis. Ophthalmology.

[2] Davis JL, Gass JDM, Pepose JS, Holland GN, Wilhelmus KR (1996). Diffuse unilateral subacute neuroretinitis. Ocular infection and immunity.

[3] Gass JDM (1987). Stereoscopic atlas of macular diseases. Diagnosis and treatment.

[4] Goldberg MA, Kazacos KR, Boyce WM, Ai E, Katz B (1993). Diffuse unilateral subacute neuroretinitis: morphometric, serologic, and epidemiologic support for Baylisascaris as a causative agent. Ophthalmology.

[5] Küchle M, Knorr HL, Medenblik-Frysch S, Weber A, Bauer C, Naumann GO (1993). Diffuse unilateral subacute neuroretinitis syndrome in a German most likely caused by the raccoon roundworm, Baylisascaris procyonis. Graefes Arch Clin Exp Ophthalmol.

[6] Souza EC, Cunha S, Gass JD (1992). Diffuse unilateral subacute neuroretinitis in south america. Arch Ophthalmol.

[7] Oueghlani E, O’Sullivan E, Pavesio CE (2010). Diffuse unilateral subacute neuroretinitis in the United Kingdom. Int Ophthalmol.

[8] de Souza EC (1999). Diffuse bilateral subacute neuroretinitis. Arch Ophthalmol.

[9] de Amorim Garcia Filho CA, Gomes AHB, Garcia ACMDA, de Amorim Garcia CA (2012). Clinical features of 121 patients with diffuse unilateral subacute neuroretinitis. Am J Ophthalmol.

[10] Berbel RF, Casella AMB, de Souza EC, Farah ME (2014). Evaluation of patients with diffuse unilateral subacute neuroretinitis by spectral domain optical coherence tomography with enhanced depth imaging. Clin Ophthalmol.

[11] Roy FH, Fraunfelder FW, Fraunfelder FT (2008). Roy and Fraunfelder’s current ocular therapy.

[12] Sabrosa NA, Arevalo JF (2010). DUSN: a potentially blinding parasitic infection. Rev Ophthalmol.

[13] Matsumoto BT, Adelberg DA, Del LP (1995). Transretinal membrane formation in diffuse unilateral subacute neuroretinitis. Retina.

[14] Graeff-Teixeira C, Loureiro Morassutti A, Kazacos KR (2016). Update on baylisascariasis, a highly pathogenic zoonotic infection. Clin Microbiol Rev.

[15] Kazacos KR, Jelicks LA, Tanowitz HB (2013). Handbook of clinical neurology.

[16] Vedantham V, Vats MM, Kakade SJ, Ramasamy K (2006). Diffuse unilateral subacute neuroretinitis with unusual findings. Am J Ophthalmol.

[17] Venkatesan P (1998). Albendazole. J Antimicrob Chemother.

